# Dapagliflozin alleviates cardiac fibrosis through suppressing EndMT and fibroblast activation via AMPKα/TGF‐β/Smad signalling in type 2 diabetic rats

**DOI:** 10.1111/jcmm.16601

**Published:** 2021-06-25

**Authors:** Jingjing Tian, Mingjun Zhang, Mengying Suo, Dian Liu, Xuyang Wang, Ming Liu, Jinyu Pan, Tao Jin, Fengshuang An

**Affiliations:** ^1^ The Key Laboratory of Cardiovascular Remodeling and Function Research Chinese Ministry of Education Chinese National Health Commission and Chinese Academy of Medical Sciences The State and Shandong Province Joint Key Laboratory of Translational Cardiovascular Medicine Qilu Hospital of Shandong University Jinan China; ^2^ Department of Cardiology The First Affiliated Hospital Zhejiang University School of Medicine Zhejiang China; ^3^ Department of Cardiology Shandong Provincial Qianfoshan Hospital of Shandong First Medical University Jinan China

**Keywords:** cardiac fibroblast, cardiac fibrosis, dapagliflozin, diabetic cardiomyopathy, endothelial‐to‐mesenchymal transition, oxidative stress, SGLT2 inhibitor

## Abstract

Diabetic cardiomyopathy (DCM) is one of the leading causes of heart failure in patients with diabetes mellitus, with limited effective treatments. The cardioprotective effects of sodium‐glucose cotransporter 2(SGLT2) inhibitors have been supported by amounts of clinical trials, which largely fills the gap. However, the underlying mechanism still needs to be further explored, especially in terms of its protection against cardiac fibrosis, a crucial pathophysiological process during the development of DCM. Besides, endothelial‐to‐mesenchymal transition (EndMT) has been reported to play a pivotal role in fibroblast multiplication and cardiac fibrosis. This study aimed to evaluate the effect of SGLT2 inhibitor dapagliflozin (DAPA) on DCM especially for cardiac fibrosis and explore the underlying mechanism. In vivo, the model of type 2 diabetic rats was built with high‐fat feeding and streptozotocin injection. Untreated diabetic rats showed cardiac dysfunction, increased myocardial fibrosis and EndMT, which was attenuated after treatment with DAPA and metformin. In vitro, HUVECs and primary cardiac fibroblasts were treated with DAPA and exposed to high glucose (HG). HG‐induced EndMT in HUVECs and collagen secretion of fibroblasts were markedly inhibited by DAPA. Up‐regulation of TGF‐β/Smad signalling and activity inhibition of AMPKα were also reversed by DAPA treatment. Then, AMPKα siRNA and compound C abrogated the anti‐EndMT effects of DAPA in HUVECs. From above all, our study implied that DAPA can protect against DCM and myocardial fibrosis through suppressing fibroblast activation and EndMT via AMPKα‐mediated inhibition of TGF‐β/Smad signalling.

## 1 INTRODUCTION

Diabetes mellitus (DM) describes a group of metabolic disorders featured by persistent hyperglycaemia, which has constituted one of the leading causes of death globally according to the report by WHO. The increasingly rising prevalence has been predicted by the International Diabetes Federation (IDF) to reach 10.4% (642 million) by 2040.^1^ Cardiovascular diseases are by far recognized as the major cause of morbidity and mortality among people with diabetes mellitus.^2^ Despite that macrovascular events play a leading role in diabetic cardiac complications, heart failure in the absence of coronary artery disease, hypertension and significant valvular disease is common as well in diabetes, which is referred to as diabetic cardiomyopathy (DCM).^3^, ^4^ In terms of the complicated and interrelated pathogenesis of DCM, studies have reported that hyperglycaemia and metabolic disturbance in diabetes can give rise to cardiomyocyte apoptosis, deposition of extracellular matrix (ECM), endothelial dysfunction and oxidative stress, thus leading to the development of DCM.^5^, ^6^ Moreover, prolonged exposure to oxidative stress results in chronic inflammation and cardiac fibrosis.^7^ In particular, ECM deposition and cross‐linking lead to cardiac fibrosis and stiffness, which are closely associated with the primary pathophysiological features of DCM, namely ventricular remodelling, diastolic dysfunction and contractility impairment.^4^, ^8^

During the process of cardiac fibrosis, cardiac fibroblasts (CFs) are the main source of ECM.^6^ Besides, in the pathological course of DCM, endothelial cells are the headmost targets of hyperglycaemia, and endothelial‐to‐mesenchymal transition (EndMT) is an important cellular phenotype shift that multiplies CFs and aggravates cardiac fibrosis.^9^, ^10^ Hence, fibroblast‐like cells derived from endothelial cells via EndMT play a crucial role in diabetic cardiac fibrosis.^11^ In the process of transdifferentiation, endothelial cells lose endothelial markers such as CD31 and vascular endothelial cadherin (VE‐cadherin) and gain mesenchymal features such as α‐smooth muscle actin (α‐SMA), Vimentin and fibroblast‐specific protein‐1(FSP‐1) accompanied by the up‐regulated transcription level of EndMT markers (snial1, snial2, twist1 and twist2).^12^, ^13^, ^14^ Concerning molecular mechanisms, transforming growth factor‐β (TGF‐β)/Smad signalling plays a vital role in the pathogenesis of cardiac fibrosis and is the utmost pathway to mediate EndMT.^15^, ^16^

The heavy financial burden and serious health threat posed by diabetic cardiac complications lead to an urgent need for effective treatment. However, classical glucose‐lowering therapies generally have a neutral effect on cardiovascular mortality in diabetes and at times even aggravate heart failure.^17^ Sodium‐glucose cotransporter‐2 (SGLT2) inhibitors, a new class of antidiabetic drugs approved by the FDA in 2013, act directly on the SGLT2 receptor of proximal tubule of the kidney and decrease renal glucose reabsorption.^18^ Based on extensive clinical trials and 2019 ESC guidelines, SGLT2 inhibitors have been given priority in treating diabetic patients with cardiovascular diseases.^19^, ^20^, ^21^ Importantly, the protective effect of SGLT2 inhibitors against heart failure has been found to act independently of glucose control,^22^ whereas the underlying mechanism is not yet fully elucidated. Previous studies generally focused on inflammation and oxidative stress involved in the cardioprotective effect of SGLT2 inhibitors in diabetes,^23^, ^24^, ^25^ with limited mechanism exploration into cardiac fibrosis alleviation.

The present study aimed to evaluate the effect of SGLT2 inhibitor dapagliflozin (DAPA) on DCM, especially cardiac fibrosis and EndMT in a rat model of type 2 diabetes. Cellular phenotype and molecule mechanisms were further explored in HG‐stimulated HUVECs and CFs.

## 2 MATERIALS AND METHODS

### 2.1 Animals

We purchased sixty 4‐week‐old male Sprague Dawley (SD) rats from Beijing Weitong Lihua Experimental Animal Technology and place them in the environment accessible to feed and water with an alternate 12‐hour day‐night cycle at 22 ± 2°C. After 1‐week adaptive feeding, all rats were randomized to four groups (n = 15 for each group). Rats in the control group were fed with normal chow and the other three groups with high‐fat feed (16% fat and 0.30% cholesterol). Four weeks later, intraperitoneal insulin tolerance test (IPITT) and intraperitoneal glucose tolerance test (IPGTT) were performed to identify rats with insulin resistance, which were then injected intraperitoneally once with streptozotocin (STZ; 35 mg/kg, Solarbio) to produce the type 2 diabetic models. One week after the injection, we took caudal vein blood samples to measure fasting blood glucose (FBG). Only rats with FBG ≥11.1 mmol/L were regarded as a successful type 2 diabetic model and selected for further investigation. After another 8 weeks of the high‐fat diet, two groups of diabetic rats were respectively given dapagliflozin (DAPA, 1 mg/kg·day) and metformin (MET, 200 mg/kg·day) in the drinking water. Adjustment of the concentration of DAPA and metformin was made accordingly every 4 days based on the water intake. Eight weeks later, IPITT and IPGTT were performed and all rats were killed with bodyweight and FGB measured. In brief, rats were divided into four groups: Control, DCM, DCM +DAPA and DCM +MET. We took the animal care and experimental procedures following the Guidelines for the Care and Use of Laboratory Animals approved by Shandong University Animal Care Committee and Institutional Ethics Committee of Shandong University.

### 2.2 Cardiac function

Rats were initially anaesthetized through inhalation of isoflurane (2.5%) and kept under general anaesthesia with persistent 1.5% isoflurane provided by a nasal tube connected to the anaesthesia machine. The cardiac function of rats was evaluated by echocardiography using the Vevo 770 imaging system with RMB710 transducer (VisualSonics). The left ventricle ejection fraction (LVEF), fractional shortening (FS), early‐to‐late mitral diastolic flow ratio (E/A) and ratio of diastolic mitral annulus velocities (E′/A′) were measured.

### 2.3 Histology and immunohistochemistry

Rat heart tissues were fixed with formalin, embedded in paraffin and subsequently cut into 5 μm sections. Heart sections were stained with haematoxylin and eosin (HE) to exhibit cardiac structure. Masson's trichrome and Picrosirius red staining were carried out to detect collagen deposition. The quantitative analysis of the cardiomyocyte diameter and collagen volume was conducted with IPP (Image‐Pro Plus) software in randomly chosen areas (200×). Heat‐induced antigen retrieval was performed followed by blocking with 5% BSA and incubation at 4°C overnight with primary antibodies against collagen III (Abcam, ab7778), collagen I (Abcam, ab34710), VE‐cadherin (Abcam, ab231227), Vimentin (CST, #5741), FSP‐1 (Abcam, ab197896) and NOX4 (Proteintech, 14347‐1‐AP) for immunohistochemical analysis, or CD31 (Novus, NB100‐2284) and α‐SMA (Sigma, A2547) for immunofluorescence co‐staining in paraffin‐embedded sections. Thereafter, the samples were incubated with HRP‐conjugated or fluorescent secondary antibodies at 37°C for 30 minutes followed by DAB staining or fluorescence microscope observation. Pannoramic SCAN (3D HISTECH) was used to obtain images of slides.

### 2.4 Cell treatment

Human umbilical vein endothelial cells (HUVECs; ATCC) were cultured in the six‐well plate (Corning) with endothelial cell medium (5.5 mM glucose, ScienCell) supplemented with 5% foetal bovine serum, 1% penicillin‐streptomycin and 1% endothelial cell growth supplement (ECGS) in the 5% CO_2_ thermostatic (37°C) incubator for more than 12 hours. When the cell density reached 70%, HUVECs were subject to 6‐hour serum starvation and pre‐treatment of DAPA (dissolved in DMSO) before exposure to high glucose (HG, 33.3 mM). Thereafter, cells were cultured under HG for at least 48h in the presence or absence of different concentrations of DAPA (0.1, 0.5, 1 μM) with DMSO treatment as a control (vehicle group). Cells under the normal glucose (NG, 5.5 mM) group were exposed to 27.8 mM mannitol to balance the osmotic pressure. To figure out the role of AMPKα, compound C (2 μM, Sigma‐171260) and AMPKα siRNA (Santa Cruz‐sc45312) were used for inhibition, with AICAR (2 mM, Sigma‐A9978) and A‐769662(10 μM, MCE‐HY50662) for activation. As also, HUVECs were pre‐treated with the reagents mentioned above before HG treatment. RNA interference was performed in Opti‐MEM reduced‐serum medium (Gibco Life Technologies) for 6 hours conforming to the instructions of lipofectamine 2000 (Invitrogen). HUVECs from passage 2‐5 were used for experiments.

Primary cardiac fibroblast (CFs) was isolated from newborn SD rats (2‐3 days). The first step was to separate out the ventricular tissues for subsequent digestion with collagenase type II (Solar bio). Then, cells obtained from digestion were placed in Dulbecco's modified Eagle's medium (DMEM, 5.5 mM glucose and 10% FBS) and seeded in six‐well culture plates. When the cell density reached 70%, the medium was replaced by FBS‐free DMEM for 6‐hour serum starvation. After the pre‐treatment of DAPA, cells were incubated under normal glucose (NG, 5.5 mM) or high glucose (HG, 33.3 mM) in the presence or absence of different concentrations of DAPA (0.1, 0.5, 1, 5 μM) for 48 hours.

### 2.5 Immunofluorescence microscopy

The expression and the localization of different target proteins were observed using immunofluorescence methods. HUVECs and CFs were fixed with 4% paraformaldehyde, permeabilized and then blocked with 2% BSA. Thereafter, cells were incubated with primary antibodies against CD31 (Abcam, ab28364) and α‐SMA (Abcam, ab5694) in HUVECs, and α‐SMA (Abcam, ab5694) in CFs overnight at 4°C. After incubation with two different fluorescent secondary antibodies for 30 minutes at 37°C and staining with DAPI, cell slides were sealed using antifade mounting medium. Finally, images were obtained with immunofluorescence microscopy (Nikon).

### 2.6 Determination of cell viability and proliferation rate by CCK‐8 assay

The cell viability of HUVECs and proliferation rate of CFs were detected using the Cell Counting Kit‐8 (CCK‐8; Dojin Laboratories). Cells were seeded into 96‐well plates at an initial density of 2.5‐3 × 10^4^ cells/well with a group of blank control wells (without cells) and a group of untreated control wells (cells only treated with the medium). Each group was made in eight replicates simultaneously. After treatment with different concentrations of DAPA under HG condition for 24 hours, 10 μl kit reagent was added into 100 μl medium per well in the plate which was thereafter incubated at 37°C for 3 hours. OD values were obtained using a multimode microplate reader for the absorbance reading at 450 nm.

### 2.7 Measurement of intracellular ROS

Oxidative stress was evaluated via detecting the production of intracellular ROS in cultured HUVECs after different treatments. Cells were incubated with 10 µM 2′,7′‐dichlorofluorescein‐diacetate (DCFH‐DA, Solarbio) for 30 minutes and then washed three times with phosphate‐buffered saline for removing residual DCFH probe. The fluorescence intensity was observed under an inverted fluorescence microscope (Nikon) with the excitation and emission wavelength respectively at 488 and 525 nm.

### 2.8 Western blot

Heart tissue or cells were lysed with radioimmunoprecipitation (RIPA) lysis buffer. The prepared protein sample was separated through 10% SDS‐PAGE and then transferred to PVDF membrane (Millipore). 5% fat‐free milk was used for 1‐hour blocking at room temperature. The membrane was subsequently incubated with primary antibodies overnight at 4°C. The following day secondary antibodies were used for 1‐hour incubation at room temperature before the enhanced chemiluminescence (Millipore) with Amersham Imager 680 (General Electric Company). Primary antibodies included those against CD31 (Abcam, ab28364; Novus, NB100‐2284), VE‐cadherin (Abcam, ab231227; ab33168), α‐SMA (Abcam, ab5694), Vimentin (CST, #5741), collagen I (Abcam, ab34710), collagen III (Abcam, ab7778), Phospho‐AMPKα (Thr172) (CST, #2535), AMPKα (CST, #5831), Smad4 (CST, #38454), TGF‐β (Santa Cruz, sc130348), NOX4 (Proteintech, 14347‐1‐AP) and GAPDH (Proteintech, 10494‐1‐AP).

### 2.9 RNA extraction and RT‐PCR

We extracted the total RNA from frozen rat cardiac tissue and cells with TRIZOL^™^ (Roche Diagnostics), which was later reverse‐transcribed into cDNA using oligo(dT) primers and the PrimeScript™RT reagent Kit with gDNA Eraser (Takara). Quantitative Real‐time PCR was performed using the SYBR Premix Ex Taq Kit (TaKaRa) according to the manufacturer's protocol to determine the expression of mRNA of collagen I, collagen III, TGF‐β, CTGF, snial1, snial2, twist1 and twist2. Each reaction was conducted in triplicate and the results were normalized against GAPDH. The sequences of the oligonucleotide primers are shown in Table 1.

**TABLE 1:** All primers used in our study

Primers	Forward	Reverse
Collagen I^r^	CACTGCAAGAACAGCGTAGC	AAGTTCCGGTGTGACTCGTG
Collagen III^r^	CAGCCTTCTACACCTGCTCC	GTCGCCATTTCTCCCAGGAA
TGF‐β^r^	TGACATGAACCGACCCTTCC	TGTGGAGCTGAAGCAGTAGT
CTGF^r^	TAGCTGCCTACCGACTGGAA	CTTAGAACAGGCGCTCCACT
Snail1^r^	ACCCTCATCTGGGACTCTCTC	CTGGGAGCTTTTGCCACTGT
Snail2^r^	AGAAGCCCAACTACAGCGAA	TAAGAGGAGAAAGGCCGCTG
Twist1^r^	TCGCTGAACGAGGCATTTG	AATCTTGCTCAGCTTGTCCG
Twist2^r^	AAATCGAGCGAAGATGGCAG	CTTGTCAGAGGGGAGCGTG
β‐actin^r^	CTCTGTGTGGATTGGTGGCT	CGCAGCTCAGTAACAGTCCG
Snail1^h^	GACCCCAATCGGAAGCCTAA	AGGGCTGCTGGAAGGTAAAC
Snail2^h^	GCCAAACTACAGCGAACTGG	GATGGGGCTGTATGCTCCTG
Twist1^h^	GGACAGTGATTCCCAGACGG	CCTTTCAGTGGCTGATTGGC
Twist2^h^	ACAGCAAGAAGTCGAGCGAA	GCAGCGTGGGGATGATCTTG
β‐actin^h^	CATGTACGTTGCTATCCAGGC	CTCCTTAATGTCACGCACGAT

Note

Sequences are listed 5'–3'. r Primers used in rat experiments. h Primers used for HUVECs.

### 2.10 Statistical analysis

All data analysis was performed with Prism 8.0 (GraphPad) and SPSS 20.0. The difference comparison among groups was conducted using one‐way ANOVA with subsequent Tukey's test, Scheffe's test, Bonferroni's post hoc test or Dunnett's multiple‐to‐one comparison test. Each experiment was repeated at least 3 times, and data were shown as means ± standard deviation (SD). *P* < .05 was considered statistically significant.

## 3 RESULTS

### 3.1 Dapagliflozin attenuates left ventricular remodelling and improves cardiac function in diabetic rats

**FIGURE 1:**
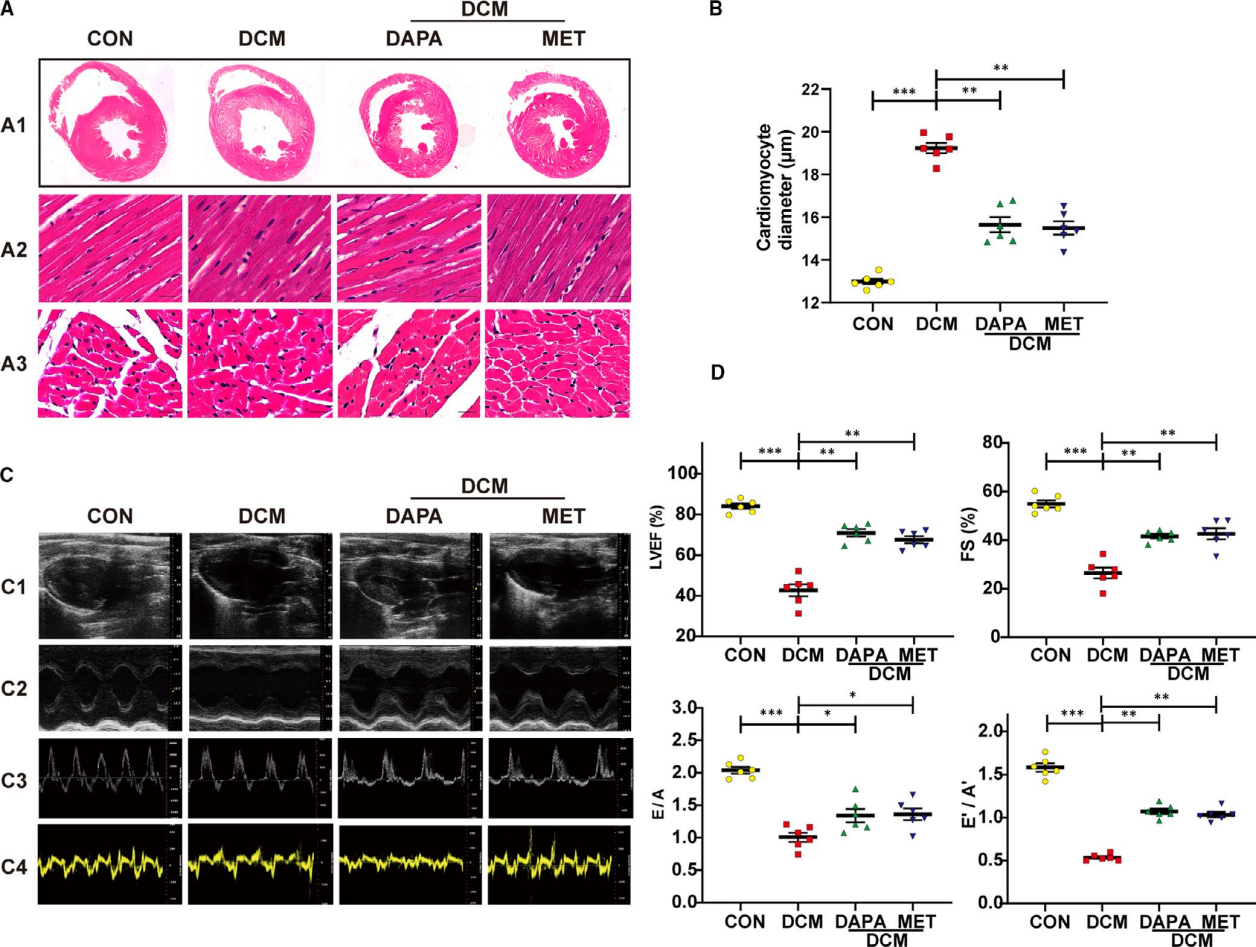
Dapagliflozin attenuates left ventricular remodelling and improves cardiac function in diabetic rats. A, Representative haematoxylin and eosin staining (HE) staining images of the cross shaft of heart at musculi papillares (A1), longitudinal left ventricular (LV) sections (A2, scale bar: 20 μm), transverse LV sections (A3, scale bar: 20 μm). B, Quantitative analysis of the cardiomyocyte diameter in heart tissue. C, Representative images of 2D echocardiograms (C1), M‐mode echocardiograms (C2), Pulse‐wave Doppler echocardiograms of mitral inflow (C3), tissue Doppler echocardiograms (C4). D, Cardiac function assessment including the left ventricle ejection fraction (LVEF), fractional shortening (FS), early‐to‐late mitral diastolic flow ratio (E/A) and ratio of diastolic mitral annulus velocities (E′/A′). DCM: diabetic cardiomyopathy; DAPA: Dapagliflozin, 1 mg/kg·day; MET: metformin, 200 mg/kg·day. (*P < .05, **P < .01, ***P < .001; Data = means ± SD; n = 6 in each group; statistical analysis was carried out using one‐way ANOVA)

Insulin resistance combined with STZ injection contributed to type 2 diabetes in rats, which were then randomized to an untreated group and groups treated with DAPA or metformin (used as a positive antidiabetic control given its confirmed cardiac protective benefits in experimental animals). Compared with the control group, HE staining of diabetic heart tissue revealed pathological hypertrophy which was restored by the administration of DAPA and metformin (Figure 1A). The significantly elevated ratio of heart weight to bodyweight and the increased diameter of cardiomyocytes were observed in diabetic hearts, which showed reduction after DAPA treatment (Table 2; Figure 1B).

**TABLE 2:** Basic information of rats

	CON	DCM	DCM	
			DAPA	MET
Blood glucose (mmol/L)	6.95 ± 0.1643	26.13 ± 2.189**	15.1 ± 4.226^#^	15.5 ± 4.411^#^
Bodyweight (g)	558 ± 16.93	342 ± 43.35**	432.3 ± 26.54^#^	449.3 ± 26.55^#^
Heart Weight (g)	1.578 ± 0.2464	2.066 ± 0.03249*	1.903 ± 0.08083*	1.91 ± 0.07*
HW / BW (mg/g)	3.414 ± 0.1797	4.708 ± 0.3711**	3.824 ± 0.1878^#^	3.778 ± 0.1054^#^

Note

Data are expressed as the means ± SD.

Statistical analysis was carried out using one‐way ANOVA.

Abbreviations: CON, control group; DAPA, dapagliflozin; DCM, diabetic cardiomyopathy; HW/BW, heart weight/bodyweight; MET, metformin.

* *P* < .05,

** *P* < .01 compared with the CON group.

^#^ *P* < .05 compared with the DCM group.

Echocardiography results showed the systolic and diastolic dysfunction in untreated diabetic rats, as evidenced by significantly reduced LVEF, FS, E/A ratio and E′/A′ ratio. In contrast, DAPA and metformin treatment reversed this reduction equally, suggesting that DAPA has a comparable beneficial effect on cardiac dysfunction with metformin (Figure 1C,D).

### 3.2 Dapagliflozin alleviates myocardial interstitial fibrosis and perivascular fibrosis in type 2 diabetic rats

**FIGURE 2:**
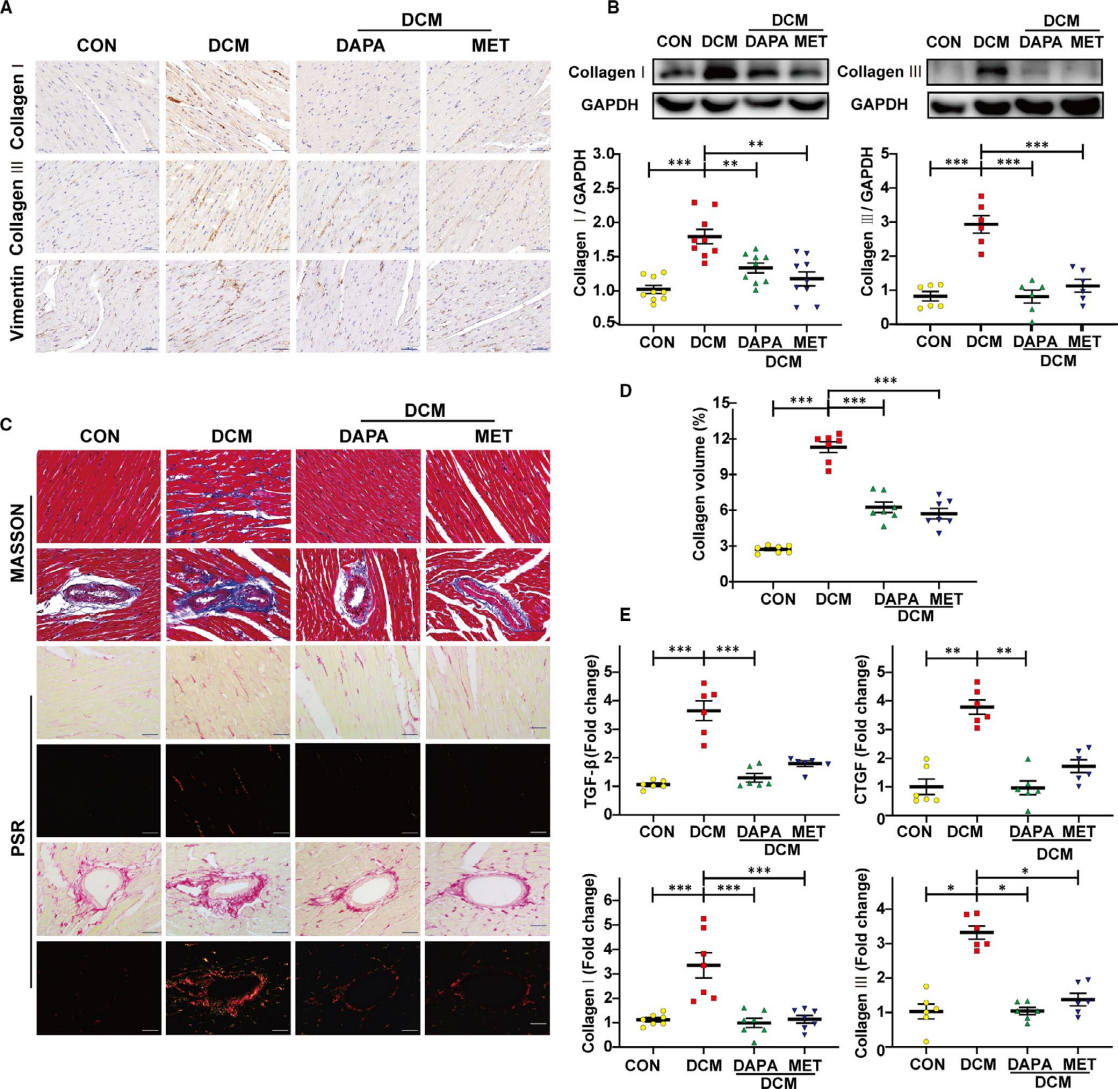
Dapagliflozin alleviates myocardial interstitial fibrosis and perivascular fibrosis in type 2 diabetic rats. A, Immunohistochemical staining of collagen I, collagen III and Vimentin (scale bar: 50 μm). B, Representative images of the Western blot of collagen I and collagen III, with the corresponding analysis. C, Masson's trichrome staining and Picrosirius red (PSR) staining of myocardial mesenchyme and peripheral vessel (scale bar: 50 μm). D, Quantification of the total LV collagen volume (PSR). E, PCR analysis of fibrotic markers (collagen I, collagen III, TGF‐β, CTGF) in diabetic heart tissue. DCM: diabetic cardiomyopathy; DAPA: dapagliflozin, 1 mg/kg·day; MET: metformin, 200 mg/kg·day. (*P < .05, **P < .01, ***P < .001; data = means ± SD; n = 6 in each group; statistical analysis was carried out using one‐way ANOVA)

Compared with the control group, Masson and Picrosirius red staining of the diabetic heart showed an increased level of collagen deposition both in the interstitial and perivascular region, whereas DAPA and metformin effectively reduced the increment (Figure 2C). Moreover, the elevated expression of collagen I, collagen III was detected with immunohistochemical staining, Western blot and mRNA quantification analyses in diabetic hearts, which was rescued after the treatment with DAPA and metformin (Figure 2A,B,E). Increased LV collagen volume and transcription level of fibrotic markers including TGF‐β and CTGF were observed in the untreated diabetic group and then reduced after DAPA treatment (Figure 2D,E).

### 3.3 Dapagliflozin inhibits cardiac EndMT in diabetic rats

**FIGURE 3:**
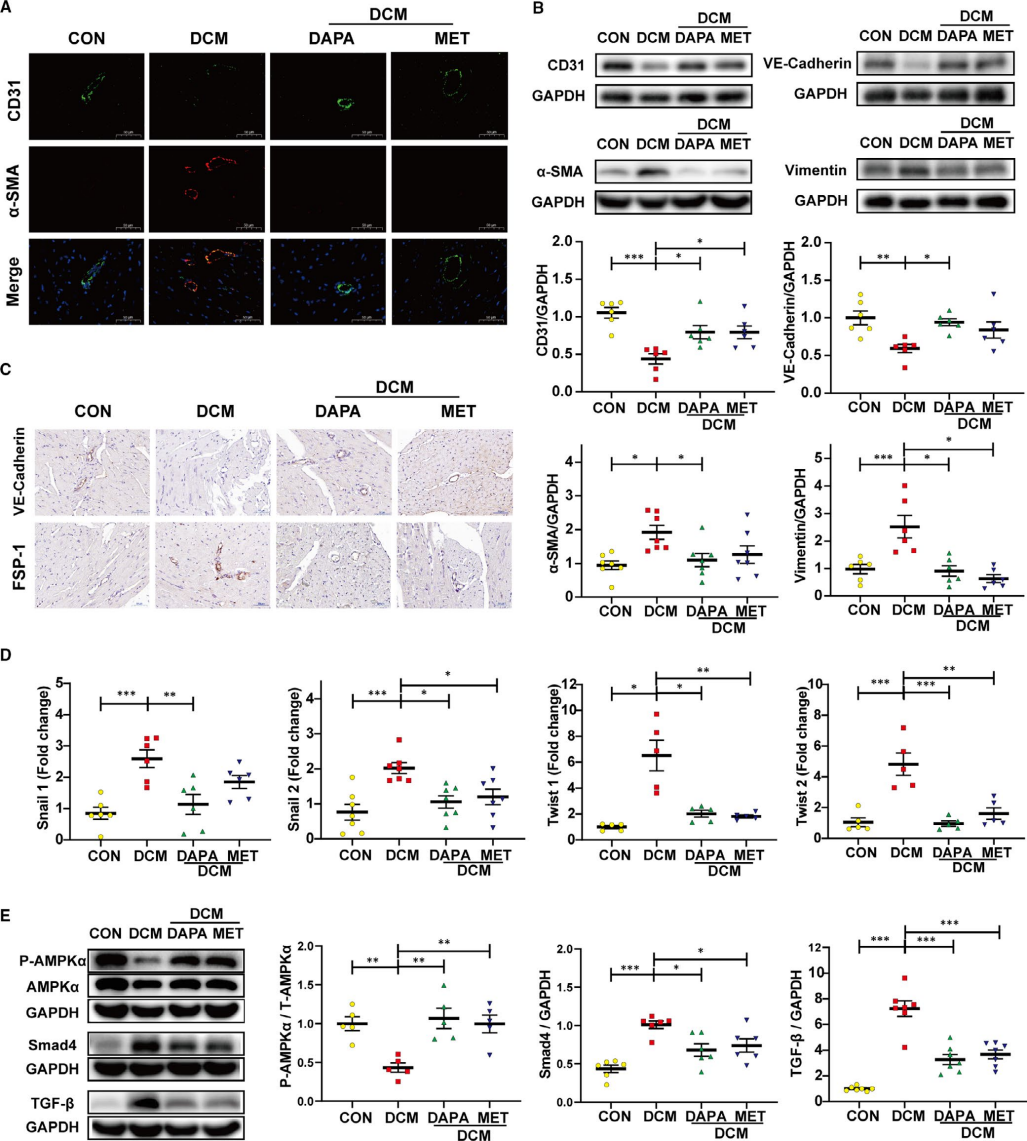
Dapagliflozin inhibits cardiac EndMT and TGFβ/Smad signalling with AMPK activation in type 2 diabetic rats. A, Immunofluorescence co‐staining of CD31 and α‐SMA (scale bar: 50 μm). B, Western blot analysis of CD31, VE‐cadherin, α‐SMA and Vimentin. C, Immunohistochemical staining of VE‐cadherin and FSP‐1 (scale bar: 50 μm). D, PCR analysis of EndMT markers (snial1, snial2, twist1, twist2). E, Western blot analysis of P‐AMPKα, AMPKα, Smad4 and TGF‐β. DCM: diabetic cardiomyopathy; EndMT, endothelial‐to‐mesenchymal transition; DAPA: dapagliflozin, 1 mg/kg·day; MET: metformin, 200 mg/kg·day. (*P < .05, **P < .01, ***P < .001; data = means ± SD; n = 6 in each group; statistical analysis was carried out using one‐way ANOVA)

In contrast with control rats, an up‐regulated expression of mesenchymal markers (α‐SMA, vimentin, FSP‐1) and down‐regulated expression of endothelial markers (CD31, VE‐cadherin) were observed in the endothelium of peripheral vessels in untreated diabetic hearts (Figure 3A‐C). The transcription level of EndMT markers (snial1, snial2, twist1, twist2) was also elevated in hearts of the diabetic rats (Figure 3D). DAPA and metformin treatment repressed the EndMT phenotype transition (Figure 3A‐D).

### 3.4 Dapagliflozin attenuates HG‐induced EndMT in HUVECs

**FIGURE 4:**
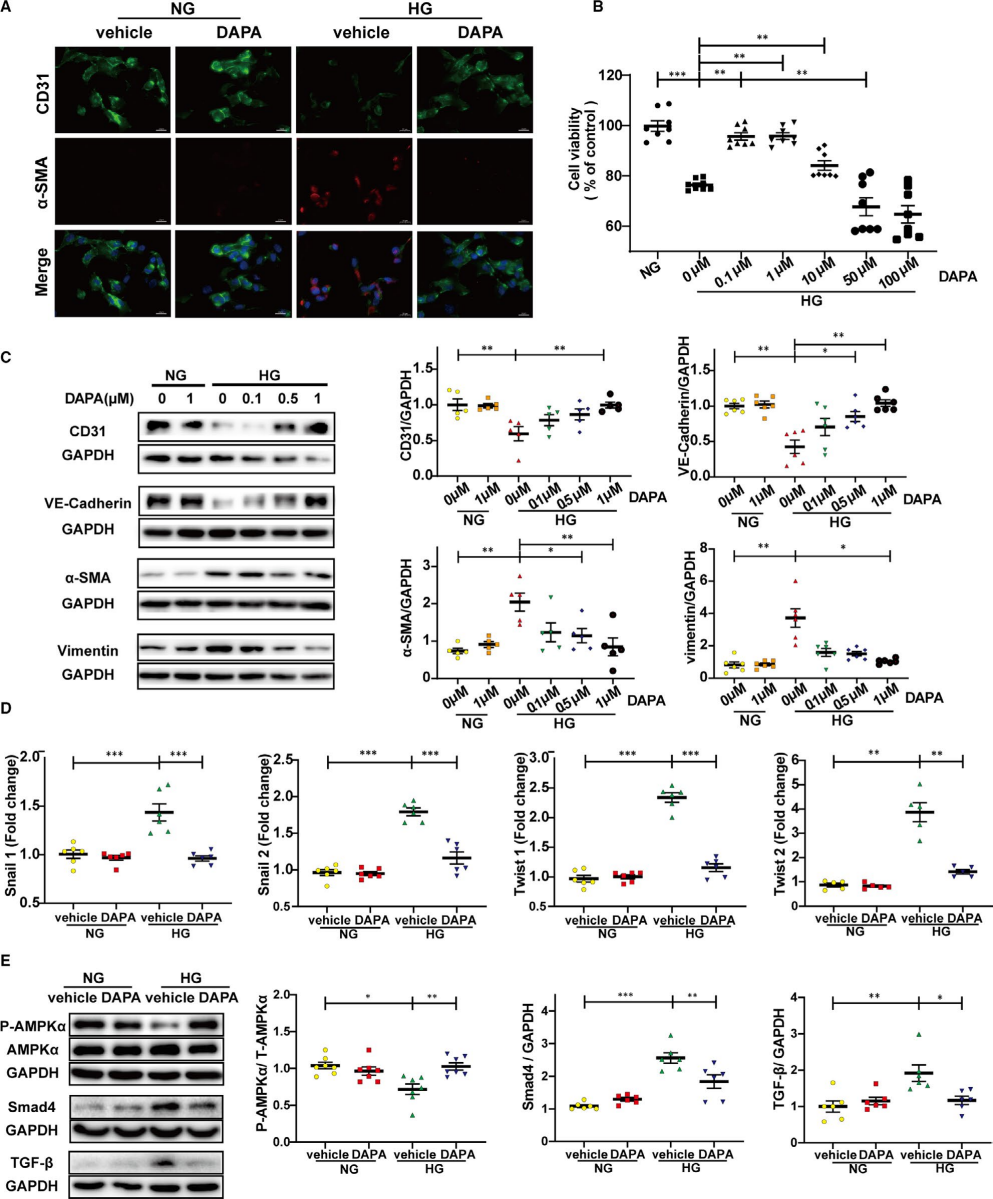
Dapagliflozin attenuates HG‐induced EndMT and TGFβ/Smad signalling with AMPKα activation in HUVECs. HUVECs were treated with DAPA or vehicle under NG or HG for at least 48 h. A, Immunofluorescence co‐staining of CD31 and α‐SMA in HUVECs (scale bar: 20 μm). B, CCK8 assay for cell viability of HUVECs treated with different concentrations of DAPA (0, 0.1, 1, 10, 50, 100 μM) under HG. C, Western blot analysis of CD31, VE‐cadherin, α‐SMA, Vimentin in HUVECs after cells were treated with DAPA (0, 0.1, 0.5, 1 μM) and exposed to HG. D, PCR analysis of EndMT markers (snial1, snial2, twist1, twist2) in HUVECs. E, Western blot analysis of P‐AMPKα, AMPKα, Smad4, TGF‐β in the indicated group. NG: normal glucose, 5.5 mM; HG: high glucose, 33.3 mM; EndMT, endothelial‐to‐mesenchymal transition; DAPA: dapagliflozin; CCK8: Cell Counting Kit‐8. (*P < .05, **P < .01, ***P < .001; Data = means ± SD; n = 6 in each group; Statistical analysis was carried out using one‐way ANOVA)

To determine the effect of DAPA on the viability of endothelial cells, HUVECs were cultured under HG in the presence of DAPA (0, 0.1, 1, 10, 50, 100 μM). CCK8 results revealed that DAPA treatment rescued the cell viability impaired by HG in a dose‐dependent manner, whereas high doses (over 10 μM) can cause cytotoxicity. Therefore, 1 μM DAPA was selected for the follow‐up experiments. (Figure 4B).

HG induced endothelial‐to‐mesenchymal phenotype transition in HUVECs, mainly manifested as up‐regulated expression of fibrotic markers (α‐SMA, vimentin), down‐regulated expression of endothelial markers (CD31, VE‐cadherin) and elevated transcription level of EndMT markers (snail1, snail2, twist1, twist2) (Figure 4A,C,D). As shown in Figure 4, the transition of losing endothelial features and acquiring mesenchymal features was reversed after DAPA treatment in dose dependence.

### 3.5 AMPKα inhibition abrogates the anti‐EndMT effects of dapagliflozin

**FIGURE 5:**
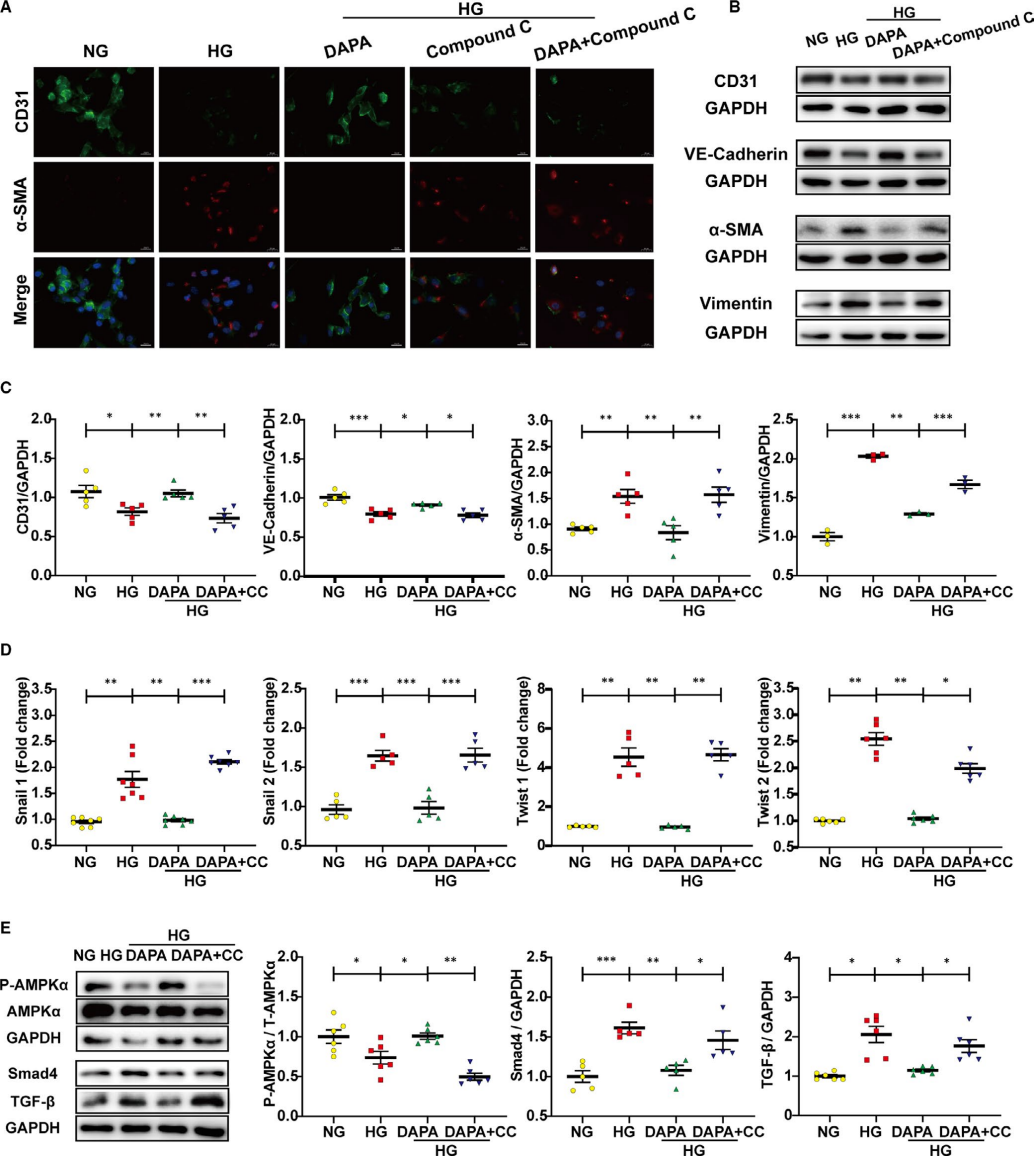
AMPKα inhibition by compound C abrogates the anti‐EndMT effects of dapagliflozin. HUVECs were treated with DAPA (1 μM) and compound C (2 μM) under HG for at least 48 h. A, Immunofluorescence co‐staining of CD31 and α‐SMA in HUVECs (scale bar: 20 μm). B, Representative images of the western blot of CD31, VE‐cadherin, α‐SMA and Vimentin, with the corresponding analysis (C). D, PCR analysis of EndMT markers (snial1, snial2, twist1, twist2) in HUVECs. E, Western blot analysis of P‐AMPKα, AMPKα, Smad4, and TGF‐β in the indicated group. NG: normal glucose, 5.5 mM; HG: high glucose, 33.3 mM; EndMT, endothelial‐to‐mesenchymal transition; DAPA: dapagliflozin; CC: Compound C. (*P < .05, **P < .01, ***P < .001; data = means ± SD; n = 5 in each group; statistical analysis was carried out using one‐way ANOVA)

To explore whether the protective effect of DAPA is reliant on AMPKα activation, cells were treated with compound C and AMPKα siRNA with the efficiency of inactivation and RNA interference shown respectively in Figures 5E and 7A,B. Results indicated that the reduced phosphorylation level of AMPKα attributed to CC counteracted the attenuation of EndMT by DAPA, as evidenced by increased expression of mesenchymal markers (α‐SMA, vimentin), lowered expression of endothelial markers (CD31, VE‐cadherin) and elevated transcription level of EndMT markers (snail1, snail2, twist1, twist2) compared with DAPA group (Figure 5A‐D). Correspondingly, after the siRNA‐mediated knockdown of AMPKα, the protection against EndMT provided by DAPA was diminished as well (Figure 7C, D). To look at it another way, DAPA treatment failed to resist EndMT in the AMPKα inhibition group, which also supported the critical role played by AMPKα in the efficacy of DAPA (Figures 5A, 7C‐D).

### 3.6 Dapagliflozin augments the anti‐EndMT effects of AMPKα agonist

**FIGURE 6:**
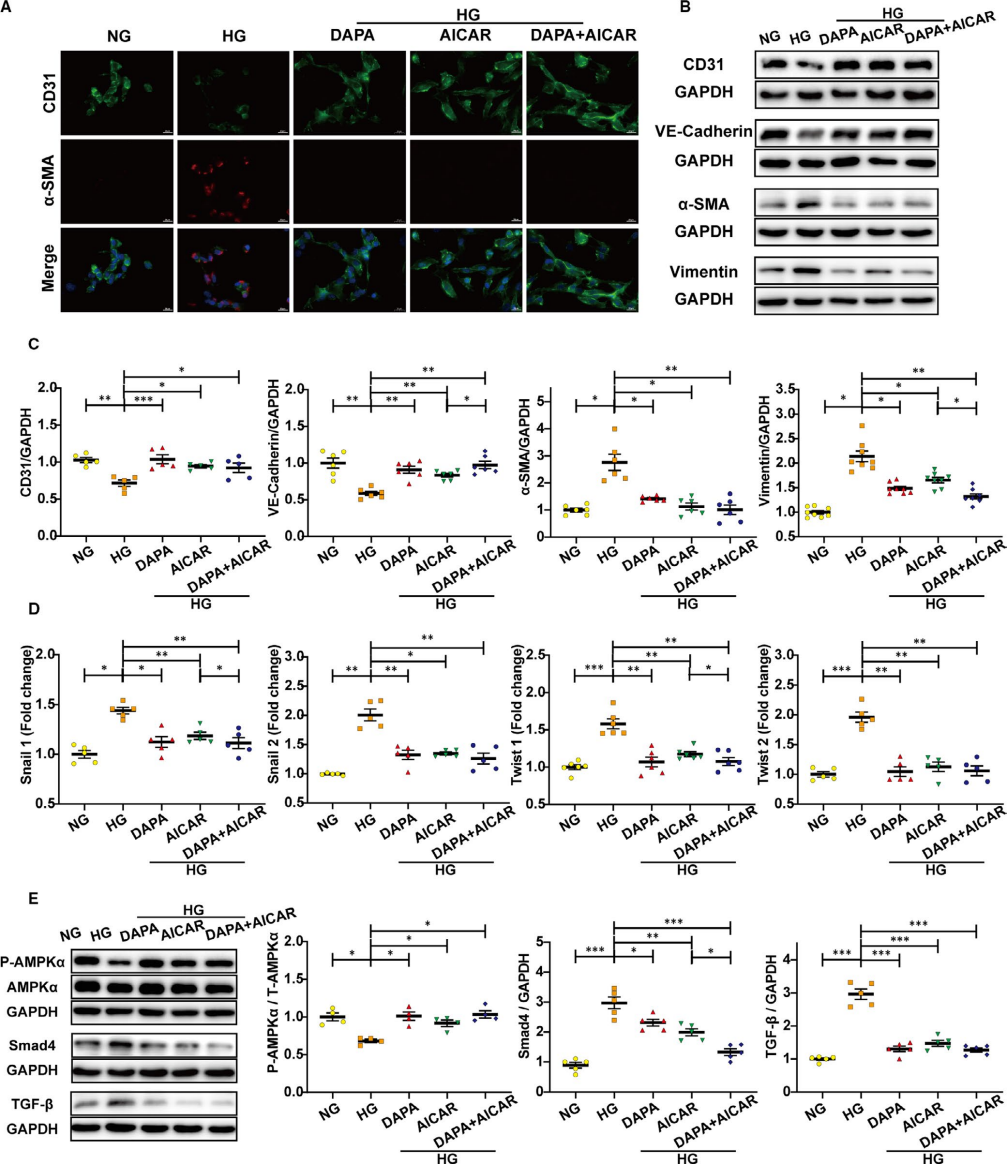
Dapagliflozin augments the anti‐EndMT effects of AMPKα agonist‐AICAR. HUVECs were treated with DAPA (1 μM) and AMPKα agonist (AICAR, 2 mM) under HG for at least 48 h. A, Immunofluorescence co‐staining of CD31 and α‐SMA in HUVECs (scale bar: 20 μm). B, Representative images of the Western blot of CD31, VE‐cadherin, α‐SMA and Vimentin, with the corresponding analysis (C). D, PCR analysis of EndMT markers (snial1, snial2, twist1, twist2) in HUVECs. (E) Western blot analysis of P‐AMPKα, AMPKα, Smad4 and TGF‐β in the indicated group. NG: normal glucose, 5.5 mM; HG: high glucose, 33.3 mM; EndMT, endothelial‐to‐mesenchymal transition; DAPA: dapagliflozin. (*P < .05, **P < .01, ***P < .001; data = means ± SD; n = 5 in each group; statistical analysis was carried out using one‐way ANOVA)

**FIGURE 7:**
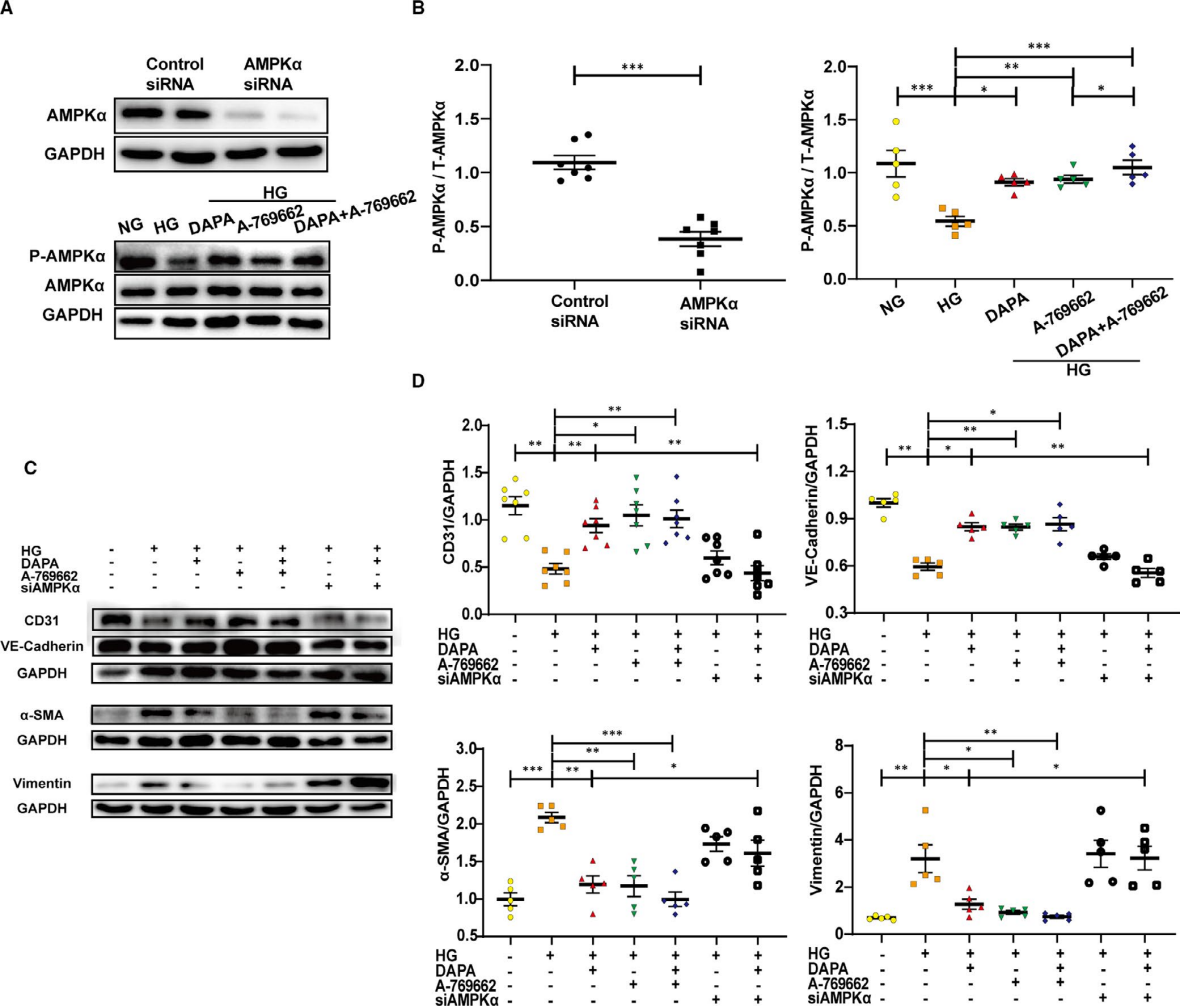
Dapagliflozin rescues HG‐induced EndMT in an AMPKα‐dependent manner in HUVECs. HUVECs were treated with DAPA (1 μM), AMPKα agonist (A‐769662, 10μM) or AMPKα siRNA under HG for at least 48 h. A, Efficiency of RNA interference and agonist assessed by the Western blot of P‐AMPKα and AMPKα, with the corresponding analysis (B). C, Representative images of the Western blot of CD31, VE‐cadherin, α‐SMA and Vimentin, with the corresponding analysis (D). NG: normal glucose, 5.5 mM; HG: high glucose, 33.3 mM; EndMT, endothelial‐to‐mesenchymal transition; DAPA: dapagliflozin. (*P < .05, **P < .01, ***P < .001; data = means ± SD; n = 5 in each group; statistical analysis was carried out using one‐way ANOVA)

We then used AMPKα agonist (AICAR and A‐769662) to explore the additive effects with DAPA. AICAR inhibited TGF‐β/smad4 activation and exerted anti‐EndMT effect with lowered expression of fibrotic markers (α‐SMA, vimentin), increased expression of endothelial markers (CD31, VE‐cadherin) and decreased transcription level of EndMT markers (snail1, snail2, twist1, twist2). DAPA addition further improved these effects when compared with that in the HG‐AICAR group (Figure 6A‐E). The same results were likewise obtained from experiments with A‐769662 (Figure 7C,D). These results further supported the key role of AMPKα activation in DAPA‐mediated anti‐EndMT inhibition.

### 3.7 Dapagliflozin inhibits TGF‐β/Smad signalling and promotes AMPKα activation in the diabetic heart and HG‐stimulated HUVECs

The reduced phosphorylation level of AMPKα was detected in both diabetic hearts (Figure 3E) and HG‐stimulated HUVECs (Figure 4E), while DAPA restored the phosphorylation level of AMPKα as effectively as metformin which is recognized as a LKB1‐AMPK activator. We then measured the TGF‐β/Smad signalling which is the most important pathway during the process of EndMT. As expected, the expression of TGF‐β and smad4 were markedly up‐regulated in both diabetic rat hearts and HG‐stimulated HUVECs, which were blocked by DAPA (1 μM) (Figures 3E, 4E).

### 3.8 Dapagliflozin rescues HG‐induced EndMT through AMPKα‐mediated inhibition of TGF‐β/Smad signalling in HUVECs

To evaluate whether TGF‐β/Smad signalling was inhibited by DAPA in an AMPKα‐dependent manner, relevant protein expression was detected in HUVECs treated with CC and AICAR. Despite the lowered expression of TGF‐β/Smad signalling and increased phosphorylation level of AMPKα after DAPA treatment, administration of CC blocked the activation of AMPKα and raised the expression of TGF‐β/Smad signalling again (Figure 5E). In addition, AICAR exerted an additional effect with DAPA on activating AMPKα and lowering TGF‐β/Smad signalling (Figure 6E). The synchronous change of TGF‐β/Smad signalling following alteration of AMPKα activity indicated that TGF‐β/Smad inhibition by DAPA was reliant on AMPKα activation.

### 3.9 Dapagliflozin inhibits the proliferation, activation and collagen production of HG‐stimulated cardiac fibroblasts

**FIGURE 8:**
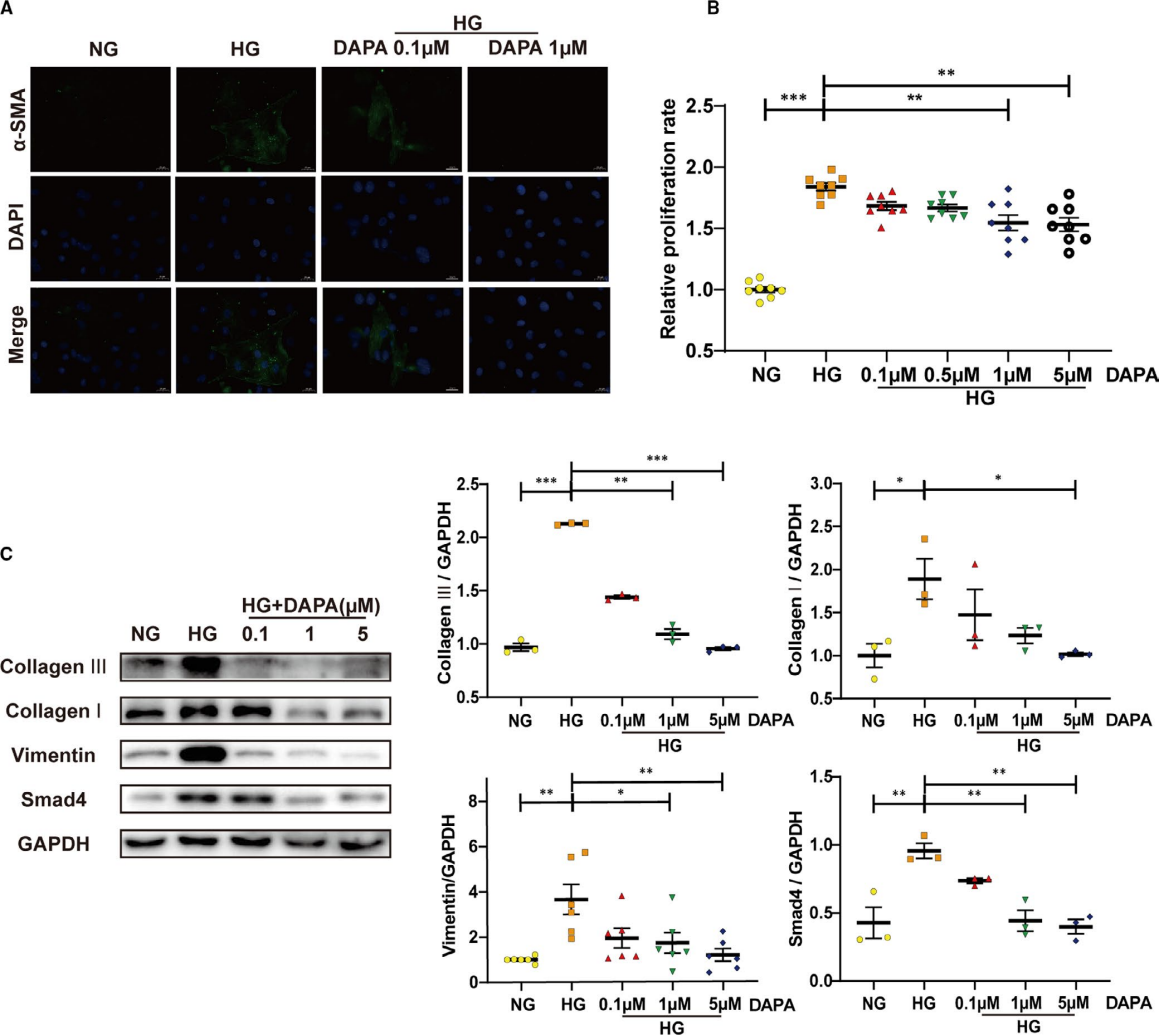
Dapagliflozin inhibits the proliferation, activation and collagen production of HG‐stimulated cardiac fibroblasts. A, Immunofluorescence staining of α‐SMA in CFs (scale bar: 20 μm). B, Relative proliferation rate of CFs treated with different concentrations of DAPA (0, 0.1, 0.5, 1, 5 μM) under HG. C, Western blot analysis of collagen I, collagen III, Vimentin and Smad4 in CFs treated with DAPA (0.1, 1, 5 μM) and exposed to HG. NG: normal glucose, 5.5 mM; HG: high glucose, 33.3 mM; CFs: cardiac fibroblasts; DAPA: dapagliflozin. (*P < .05, **P < .01, ***P < .001; data = means ± SD; n = 3 in each group; statistical analysis was carried out using one‐way ANOVA)

CFs were isolated from neonatal rats and were cultured under HG in the presence of DAPA (0, 0.1, 0.5, 1, 5 μM). CCK8 results revealed that DAPA treatment repressed the proliferation of CFs stimulated by HG in a dose‐dependent manner (Figure 8B). In addition, HG‐induced up‐regulated Smad4, α‐SMA and Vimentin expression, which was also abolished by the administration of DAPA (Figure 8A,C). As also, activated collagen synthesis function of the primary CFs was remarkably blocked by DAPA (Figure 8C).

### 3.10 Dapagliflozin ameliorates oxidative stress in the diabetic cardiac tissue and HG‐stimulated HUVECs via AMPKα activation

**FIGURE 9:**
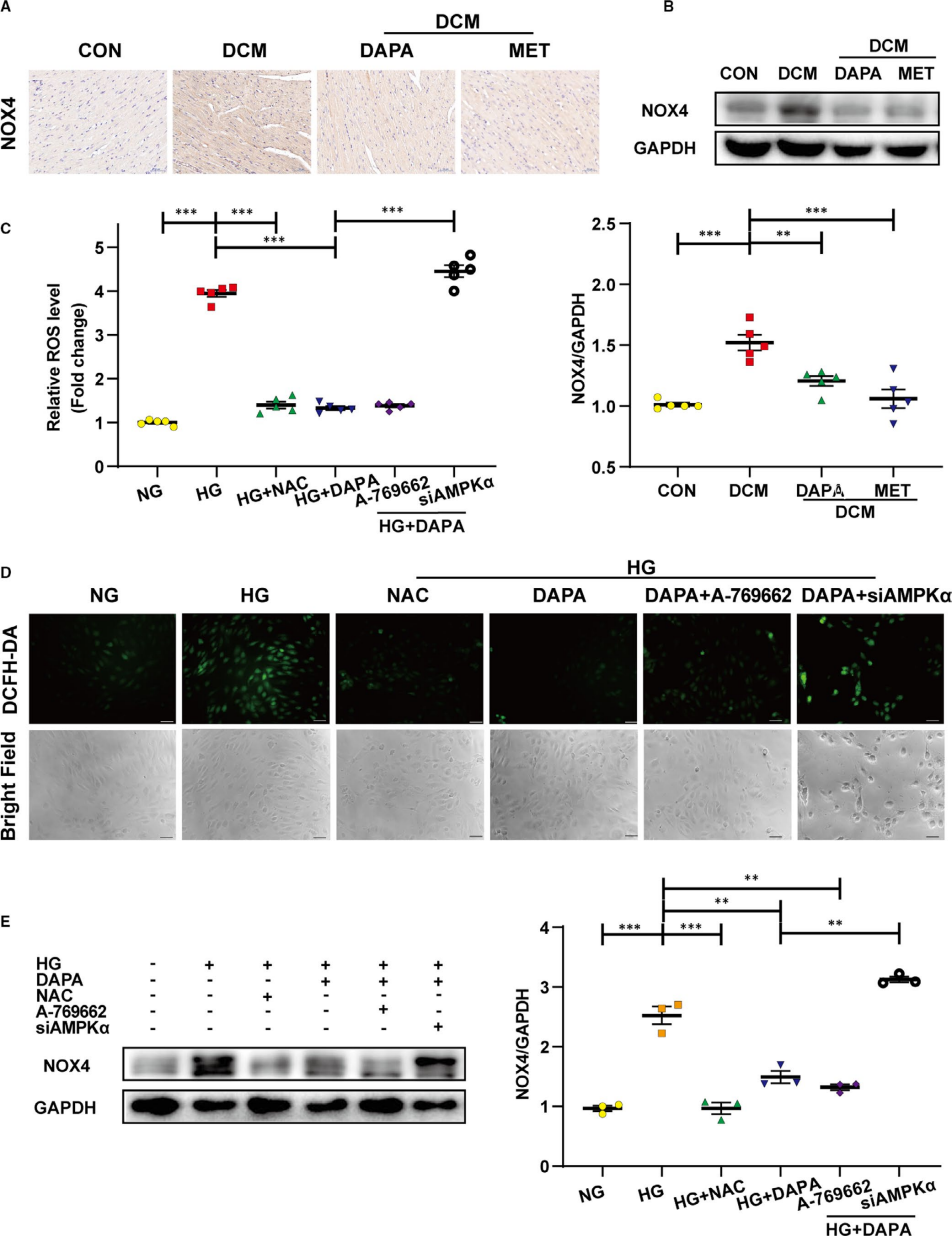
Dapagliflozin ameliorates oxidative stress in the diabetic cardiac tissue and HG‐stimulated HUVECs via AMPKα activation. A, Immunohistochemical staining of NOX4 in diabetic hearts (scale bar: 50 μm). B, Western blot analysis of NOX4 in cardiac tissues. C, Relative cellular ROS level measured with relative fluorescence intensity of DCF. D, Intracellular ROS generation in HUVECs was detected using 2′,7′‐dichlorofluorescein‐diacetate (DCFH‑DA) fluorescence probe method with subsequent observation under the fluorescence microscopy after treatment with DAPA (1 μM), A‐769662 (10 μM), siAMPKα or NAC (3 mM) under HG (scale bar: 50 μm). E, Western blot analysis of NOX4 in the indicated group of HUVECs. NG: normal glucose, 5.5 mM; HG: high glucose, 33.3 mM; DCM: diabetic cardiomyopathy; DAPA: dapagliflozin; MET: metformin; NAC: N‐acetyl cysteine; NOX4: NADPH Oxidase 4. (*P < .05, **P < .01, ***P < .001; data = means ± SD; n = 5 in each group; statistical analysis was carried out using one‐way ANOVA)

To determine the effect of DAPA on oxidative stress in diabetic rat hearts, we detected the expression of NOX4 through Western blot and immunohistochemistry. Markedly higher levels of NOX4 expression in diabetic hearts compared with the control group were reversed after DAPA and metformin treatment (Figure 9A,B). DCFH‑DA assay was then performed to detect reactive oxygen species (ROS) levels in HUVECs, showing that HG led to increased cellular ROS production, which was reduced after DAPA treatment (Figure 9C,D). However, AMPKα siRNA abolished the antioxidant effect of DAPA, indicating the AMPKα‐dependence (Figure 9C‐E).

## 4 DISCUSSION

Diabetic patients are two to three times more likely to suffer from cardiovascular disorders such as myocardial infarction, stroke and heart failure, resulting in a significantly worsening prognosis.^26^, ^27^ Due to the lack of a cardioprotective effect seen with classical antidiabetics, many studies associated with the SGLT2 inhibitor have emerged, since its approval by FDA. Prior to that time, metformin had been widely used for the treatment of type 2 diabetes and was regarded as one of the very few antidiabetics which exhibited cardioprotective effects independent of its antihyperglycaemic properties.^28^, ^29^, ^30^, ^31^ Therefore, in this study, we used metformin as a positive control in our animal experiments and compared its effect to dapagliflozin, an SGLT2 inhibitor. We found that treatment with dapagliflozin could alleviate left ventricular remodelling (Figure 1A,B) as well as improve both diastolic and systolic cardiac function (Figure 1C,D), in rats with type 2 diabetes. This is in line with clinical trials showing that various SGLT2 inhibitors (empagliflozin, canagliflozin and dapagliflozin) exhibited cardiovascular safety and benefits.^20^, ^21^, ^32^ However, a statistically significant difference between dapagliflozin and metformin regarding improvement to cardiac structure and function was not seen in this study.

Myocardial fibrosis is associated with decreased microvasculature and disruption of normal myocardial structures and is an important pathophysiological process contributing to heart failure, by increasing myocardial stiffness and reducing stroke volume.^12^ Therefore, it is of great importance to determine the impact of dapagliflozin on excessive collagen deposition in the diabetic heart and attempt to reveal its underlying mechanism of action. Previous studies have found that metformin shows antifibrotic actions^32^, ^33^, ^34^ and was therefore used in this study as a positive control. We have used wide‐ranging methodologies to detect the accumulation of extracellular matrix proteins around both the interstitial and perivascular areas, which are indicators of excessive collagen distribution in the hearts of type 2 diabetic rats (Figure 2). Dapagliflozin significantly reduced collagen volume in these diabetic hearts, demonstrating a comparable antifibrotic effect to metformin (Figure 2). In view of the glucose‐lowering effect of dapagliflozin (Table. 2), we followed up this finding with in vitro cell culture experiments to determine whether the antifibrotic action of dapagliflozin was independent of its control of glucose levels.

Cardiac fibrosis is a process mediated by the recruitment and activation of fibroblasts, approximately 27‐35% of which are of endothelial origin, derived from the process of EndMT.^12^ The fibrosis mediated by fibroblasts originating from endothelial cells contributes to the pathological process of DCM, as has been reported previously.^11^ The inhibition for EndMT/EMT by metformin has been reported in several studies,^35^, ^36^ while the action by SGLT2 inhibitors is still unclear. Our study represents the first to investigate how dapagliflozin affects EndMT and fibroblasts and to thoroughly investigate its antifibrotic mechanism of action. As shown in Figure 3A, cells exhibiting positive immunofluorescence staining for both CD31 and α‐SMA were found in the endothelium of peripheral vessels in untreated diabetic hearts, indicating that endothelial cells in diabetic tissue undergo EndMT and would therefore contribute to the total pool of cardiac fibroblasts. In contrast, this pathological transition was rescued by dapagliflozin and metformin treatment. Similar findings were seen from immunohistochemical staining, protein expression and mRNA quantification analyses using EndMT markers (Figure 3B‐D). Moreover, our in vitro experiments demonstrated a same result, with additional information for the dose dependence (Figure 4A‐D). Our in vitro results also revealed that the cardioprotective effect of dapagliflozin was independent of its glucose‐lowering effect. Furthermore, the direct attenuation of fibroblast proliferation, activation and collagen synthesis by dapagliflozin, also showed a dose dependency (Figure 8). When we assessed cell viability, we found that under HG, there was a suppression in HUVECs, but in contrast, a stimulation in CFs; whereas dapagliflozin exerted a bidirectional impact which was favourable to HUVECs but inhibitory to CFs (Figures 4B, 8B). From the above data, it is clear that dapagliflozin exerts its antifibrotic effect by blocking the fibroblast origin and directly suppressing the activation of CFs. Therefore, we feel that we have elucidated clearly the antifibrotic mechanism of action of dapagliflozin in this study.

AMP‐activated protein kinase (AMPK) is a member of the serine/threonine (Ser/Thr) kinase family, which acts as a ‘fuel gauge’ under cell stress conditions to maintain energy balance, using an α catalytic subunit as its principal functional domain.^37^ Reduced AMPKα activity has been observed in failing human and animal hearts and is closely related to cardiac fibrosis.^38^, ^39^ Additionally, previous studies have linked SGLT2 inhibitors to AMPKα activation.^40^, ^41^, ^42^ However, whether dapagliflozin attenuates cardiac fibrosis and EndMT in an AMPKα‐dependent manner remains unknown. Metformin has been widely recognized as an AMPKα agonist^33^, ^43^ and served as a positive control in our study. We observed that the phosphorylation levels of AMPKα were reduced in both diabetic hearts (Figure 3E) and HG‐stimulated HUVECs (Figure 4E) and that the restoration of AMPKα activity after dapagliflozin treatment was comparable to that of metformin, along with inhibition of cardiac fibrosis and EndMT. However, the anti‐EndMT effect of dapagliflozin was abolished when AMPKα activity was inhibited by AMPKα siRNA and compound C (Figures 5A‐D, 7C‐D), indicating that dapagliflozin may exert its antifibrotic effects in an AMPKα‐dependent manner.

It has been reported that TGF‐β plays a crucial role in cardiac fibrosis by repressing cardiac fibroblast activation and EndMT progression.^15^ However, few studies have offered evidence for the regulation of the TGF‐β/Smad pathway by SGLT2 inhibitors in cardiac tissues.^44^ In addition, studies have reported that AMPKα activation can inhibit the TGF‐β/Smad pathway.^39^, ^45^ In the current study, the up‐regulation of the TGF‐β/Smad pathway was detected in diabetic hearts and HG‐stimulated HUVECs, and this effect was inhibited by dapagliflozin and metformin treatment with AMPKα activation (Figures 3E, 4E). The synchronous change in TGF‐β/Smad signalling following altered AMPKα activity showed that this TGF‐β/Smad inhibition by dapagliflozin was reliant upon AMPKα activation (Figures 5E, 6E). Moreover, based on the close connection between Smad4 and cFs activation,^46^ inhibited Smad4 expression may account for the direct suppression of CFs activation by dapagliflozin. Besides, previous studies have suggested that cardiogenic genes are regulated through Smad4^47^ and closely related to cFs functions,^48^ from which we may infer that the expression of cardiogenic genes is involved in the downstream mechanisms of improved cardiac fibrosis by dapagliflozin.

Oxidative stress refers to a cellular state in which the production of excessive ROS exceeds the inherent capacity of antioxidants to deal with the increasing levels, leading to damage to proteins, lipids and DNA, all of which are involved in the pathological processes of various diseases including diabetes. ROS production is catalysed by a panel of enzymes and the NAPDH oxidase (NOX) family constitutes one of the major sources. NOX4 is expressed primarily in cardiomyocyte mitochondria^49^ and may function as the major catalytic component of an endothelial NADPH oxidase.^50^, ^51^ Interestingly, recent studies have found an important antioxidant effect of SGLT2 inhibitors in cardiomyocytes.^25^ In this study, we found that dapagliflozin could significantly inhibit the increased expression of NOX4 in our diabetic rat tissues and HG‐stimulated HUVECs in an AMPKα‐dependent manner (Figure 9). Therefore, dapagliflozin may be capable of alleviating DCM through the AMPKα‐mediated inhibition of oxidative stress, generated by NADPH oxidases. In addition, NOX4‐mediated ROS production appears to serve as a permissive step towards EndMT and tissue fibrosis;^52^, ^53^ thus, dapagliflozin may exert its antifibrotic and anti‐EndMT effects at least in part by the inhibition of oxidative stress and reduction of excess ROS.

SGLT2 inhibitors can act directly on the proximal tubule of the kidney, and the primary isoform of this transporter family expressed in the heart is SGLT1, with little evidence for SGLT2.^18^ In addition, a previous study indicated that SGLT2 mRNA was undetectable in cultured human pulmonary and coronary artery EC lines.^54^ However, a different study reported that SGLT2 was expressed in cultured and native ECs under pathological conditions such as hyperglycaemia and oxidative stress.^55^ In conclusion, further experiments are required to explore the expression profile of SGLT2 under pathological conditions in the heart and ECs, and to determine a role for SGLT2 inhibitors as potential cardioprotective agents by direct inhibition of its up‐regulated receptor.

## 5 CONCLUSION

**FIGURE 10:**
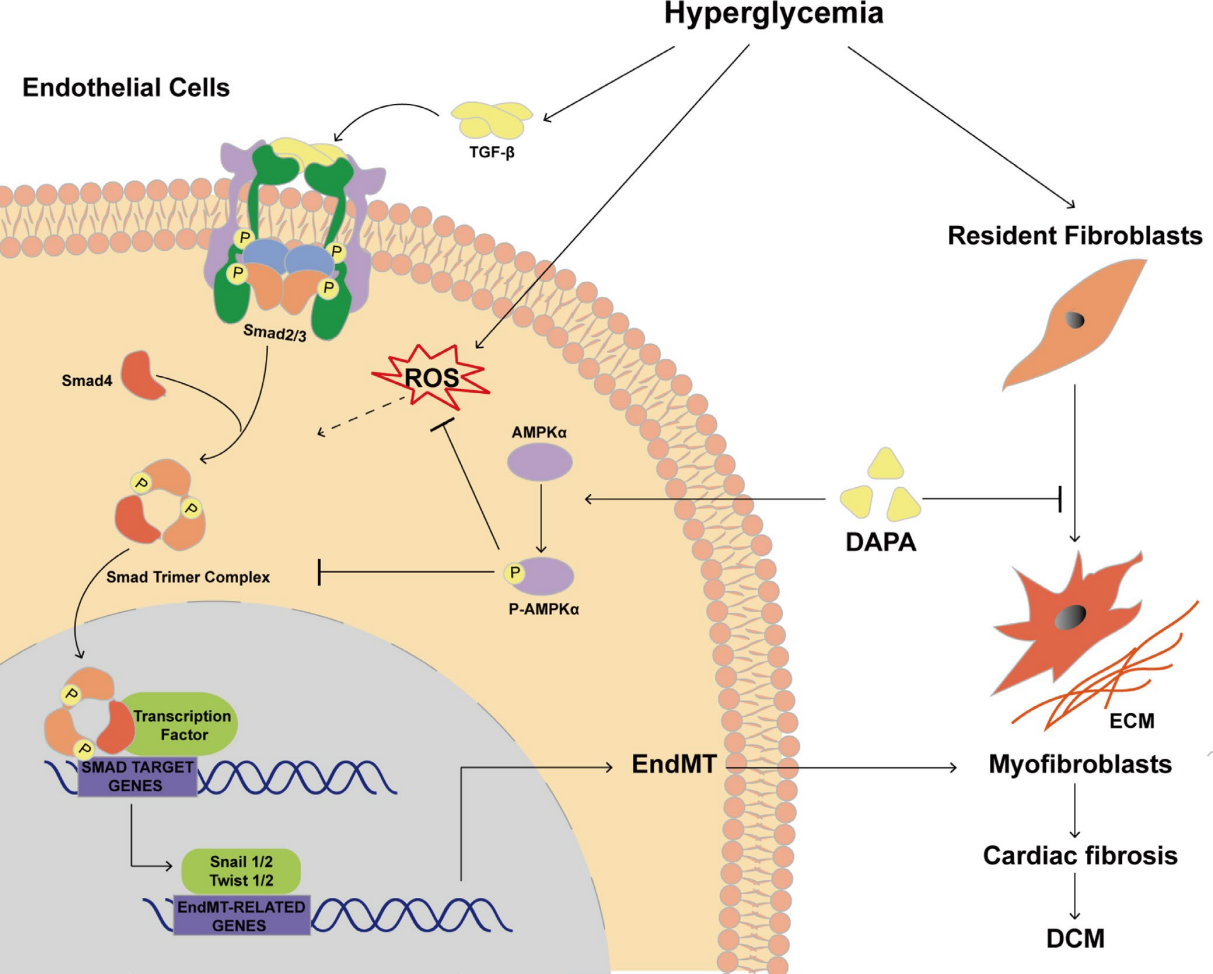
Graphical abstract. Dapagliflozin alleviates cardiac fibrosis through suppression of endothelial‐to‐mesenchymal transition and fibroblast activation via AMPKα‐mediated inhibition of TGF‐β/Smad signalling in rats with type 2 Diabetes. DAPA: dapagliflozin; ROS, reactive oxygen species; EndMT, endothelial‐to‐mesenchymal transition; ECM, extracellular matrix; DCM, diabetic cardiomyopathy

The graphical abstract of the mechanisms under the cardioprotective effect of DAPA revealed in our study is illustrated in Figure 10. Our study showed that DAPA can attenuate cardiac remodelling and ameliorate cardiac dysfunction through inhibition of fibrosis and oxidative stress in diabetic rats. As for the underlying mechanism, experiments in vitro demonstrate that DAPA can rescue HG‐induced EndMT through inhibition of TGF‐β/Smad signalling and oxidative stress in an AMPKα‐dependent manner in HUVECs. Moreover, DAPA can directly inhibit the proliferation, activation and collagen production of HG‐stimulated CFs. Therefore, our pre‐clinical observations have offered new insights into the possible mechanisms concerning cardiovascular mortality reduction by DAPA in humans, thereby providing more reliant evidence for the application of SGLT2 inhibitors. The comprehensive mechanism elucidation of the antifibrotic effect of DAPA also offers a probability for its application to other fibrotic diseases, which remains to be further explored.

## CONFLICT OF INTEREST

The authors declare that there is no conflict of interest.

## AUTHOR CONTRIBUTIONS

**Jingjing Tian:** Conceptualization (lead); Data curation (lead); Formal analysis (lead); Investigation (lead); Methodology (lead); Project administration (lead); Software (lead); Visualization (lead); Writing‐original draft (lead); Writing‐review & editing (lead). **Mingjun Zhang:** Investigation (supporting); Methodology (supporting); Project administration (supporting). **Mengying Suo:** Writing‐review & editing (supporting). **Dian Liu:** Data curation (supporting); Project administration (supporting); Writing‐review & editing (supporting). **Xuyang Wang:** Conceptualization (supporting); Formal analysis (supporting); Writing‐review & editing (supporting). **Ming Liu:** Supervision (supporting); Visualization (supporting); Writing‐review & editing (supporting). **Jinyu Pan:** Methodology (supporting); Supervision (supporting); Writing‐review & editing (supporting). **Tao Jin:** Investigation (supporting); Writing‐review & editing (supporting). **Fengshuang An:** Funding acquisition (lead); Resources (lead); Supervision (lead); Validation (lead).

## Data Availability

The data that support the findings of this study are available from the corresponding author upon reasonable request.
